# Some Properties of Composite Panels Made from Wood Flour and Recycled Polyethylene

**DOI:** 10.3390/ijms9122559

**Published:** 2008-12-10

**Authors:** Turgay Ozdemir, Fatih Mengeloglu

**Affiliations:** 1 Department of Forest Industry Engineering, Karadeniz Technical University, Trabzon, Turkey. E-Mail: tozdemir011@hotmail.com; 2 Department of Forest Industry Engineering, Kahramanmaras Sutcuimam University, Kahramanmaras, Turkey

**Keywords:** Adhesion, scratch resistance, abrasion, thickness swelling, scanning electron microscope, polyethylene, coupling agent

## Abstract

This study investigated the effect of board type (unmodified vs. MAPE modified) on the surface quality and thickness swelling-water absorption properties of recycled high density polyethylene (HDPE) based wood plastic composites. Additionally, two commercially available coatings (cellulosic coating and polyurethane lacquer coating) were also applied to composite surfaces and their adhesion strength, abrasion and scratch resistance, and gloss values were determined. This study showed that modification of the composites with MAPE coupling agent increased the surface smoothness and reduced the water absorption and thickness swelling of the panels. Abrasion resistance of the composites was also improved through MAPE modification. Regardless of board type, higher scratch resistance and gloss values were observed for polyurethane lacquer coated samples compared to those of cellulosic varnish coated ones. Improvement of adhesion strength was also seen on SEM micrographs.

## 1. Introduction

The manufacture of wood-plastic composites (WPC) utilizing recycled or virgin thermoplastics and lignocellulosic flour from wood or agricultural plants has gained significant attention over the past couple of decades [1]. This new class of materials utilizes organic fillers like wood flour instead of mica, talk, calcium carbonate etc. Organic fillers like wood flour are preferred due to the their low densities, low cost, nonabrasive nature [1, 2], possibility of high filling levels, low energy consumption, high specific properties, biodegradability and availability [3, 4].

Initially, WPC found application in automotive interior parts [1, 2, 5] and later expanded its application areas to the siding, fencing, window frames and decking markets [1–4]. Especially after their use in decking applications, manufacturing of WPCs has seen tremendous growth in the United States. Recently, WPC decking boards have gained almost 20 percent of decking market and are expected to gain more market share in the near future [4].

Several studies were conducted on the formulation of the WPCs [5–7]. Several chemicals were added to the formulation to improve the strength and durability of the materials. Even though WPC is a quite durable material, there are some issues needs to be answered like creep behavior, thermal expansion, high density, photooxidation and fading of its color [8]. Considering color changes of WPC and the use of recycled material in the production, there is a need to understand the surface and coating properties of the WPCs. However, there is not sufficient information on the coatings and adhesion properties of WPC. This study investigated the effect of maleic anhydride grafted polyethylene (MAPE) modification on the surface and thickness swelling-water absorption properties of the recycled HDPE based wood plastic composites. The effect of coating type on the adhesion strength, scratch and abrasion resistance and gloss properties of composites were also studied.

## 2. Experimental Section

### 2.1. Materials and compounding of the composites

Recycled high density polyethylene (HDPE) from used water pipes was utilized as thermoplastic matrix. The water pipes were first cleaned, cut into small pieces and then granulated into pellets. Eucalyptus wood residues from lumber mills in the city of Tarsus, Turkey were used as lignocellulosic filler. The residues were granulated into 40-mesh size flours using a Wiley mill. Maleic anhydride grafted polyethylene with an acid value of 43 mg KOH/g and density of 0.99 g/cm^3^ (Licocene PEMA 4351 by Clarient) was utilized as a coupling agent.

Two different panels were manufactured (Table 1). Depending on the groups, granulated recycled HDPE, eucalyptus wood residue (EWR) and MAPE coupling agent were mixed in a high intensity mixer for 5 minutes to produce a homogeneous blend. Then this homogenous mixture was compounded in a laboratory scale single-screw extruder at 40 rpm screw speed and at a temperature ranging from 150 to 180 °C. The extrudates were collected, cooled and granulated into 20 mesh-size pellets. Finally, pellets were compression molded in the 175 °C hot press. Press pressure and press time were 20 bar and 5 minutes, respectively. Panels of 5×150×200 mm size were produced.

### 2.2. Panel Properties

Before surface roughness measurements, the surfaces of the samples were sanded with 100 and then with 150 grid size sandpaper. A Mitutoyo Surftest SJ-301 fine stylus-type profilometer was used for surface roughness evaluation of the samples. The device consisted of a main unit and a pick-up. The pick-up has a skid-type diamond stylus with a radius of 5 μm and a tip angle of 90°. The stylus traverses the surface and its vertical displacement is converted into an electrical signal. Numerical values of surface roughness parameters can be obtained from the screen of the instrument. Cut-off length (λc) and tracing length were 2.5 and 12.5 mm, respectively. Three roughness parameters, average roughness (Ra), mean peak-to-valley height (Rz), and maximum roughness (Rmax), were used to evaluate surface roughness of the samples according to DIN 4768 [9]. Ten samples with 10 mm×10 mm dimensions were used for each test group to evaluate surface roughness. A total of 20 roughness measurements, 10 for each side of the samples, were taken.

Water absorption and thickness swelling values of the composites were determined utilizing five samples with a size of 5 cm×5 cm. Thickness swellings of the samples was calculated from the thickness measurements at five different points before and after samples were submerged in distilled water for 2, 24, 48 and 72 h. All the measurements were taken from the same location. In the case of water absorption calculations, the weight of the samples before and after submerging them in distilled water was used. Detailed description of test procedures was given by Wechsler and Hiziroglu [10].

Two different coatings which are commercially available were used in this study. These were cellulosic coating and polyurethane lacquer coating. Totals of approximately 120 g/m^2^ of varnish were sprayed onto the composite surface. Viscosity for application was 20 s, DIN cup/4mm/20 °C. Three layers of coating were applied as two bases and one topcoat layer. The component details of the coatings considered were given in Table 2. After coating, dry coating thickness was determined by using a dry film thickness apparatus [11] (Erichsen P.I.G 455). Dry film thickness of the composites was measured as 85 μ and 105 μ for cellulosic varnish and polyurethane lacquer, respectively.

Pull-off methods was used to evaluate adhesion strength between the composite surface and coating [12]. Five random measurements with a contact area of 20 mm circles were taken from each side of the samples. Erichsen Adhesion-525MC with a head glued to the surface of the samples was employed for the tests. The equipment runs a constant speed of 10 cm/min and applies tension forces to the surface layer by pulling the coating from the surface. Adhesion strength value of coating was limiting value of the tension force applied, which was registered on the display of the equipment in N/mm^2^.

Scratch resistance of the coated composites were determined by use of a scratch tester with a freely rotating supporting turntable. A special scratch diamond with a hemispherical point with 5 rpm +/- 1 rpm rotation applied to the surface. This test determines the scratch resistance as complete scratch circle at the lowest force. In this study maximum force applied was 4.0 N meaning that scratch circle was completed at lower forces than 4.0 N [13].

The taber abraser consists of a horizontal plate on which the test piece was mounted flat. The plate was turned at a frequency of 58 rpm to 62 rpm. Above the plate, two abrasive discs with rubber coating were mounted to a press that can exert a force of 5.4 N +/− 0.2N onto the test piece. Strips of sandpaper are attached to the rubber coating. The abrasion was determined by the number of rotations needed up to a certain wear-through point. The initial wear point (IP) was the point at which the first clearly recognizable wear-through of coating and the sub-layer becomes exposed. The final wear point (FP) occurred when about 95% of the coating was removed in the abraded area. The abrasion resistance was calculated based on the following formula: Wear resistance (revoluations) : (IP+FP)/2 [13].

Gloss values of the coated samples were determined using a Gardner Glossmeter. All measurements were carried out based on the standard method. During the measurement, the 60° geometry was applied to all coated samples [14]. Before the all measurements, the device was calibrated with black mirror which had 100° gloss value. Ten replicates were used for each group in order to determine the gloss values of samples.

The surface of the samples after adhesion and water soak tests was also studied by using JEOL scanning electron microscope (Model JSM 6400). Before the SEM study, the samples were mounted on the sample stub and were sputtered with gold.

Design-Expert® Version 7.0.3 statistical software program was used for statistical analysis. In this study, the general factorial design for one factor was chosen to determine effect of board type on surface roughness and water soak properties while two factor analyses was utilized to determine the effect of board type and varnish type on the abrasion, hardness and opaqueness properties of produced composites.

## 3. Results and Discussion

### 3.1. Determination of surface roughness and thickness swelling - water absorption properties of HDPE based wood plastic composites

In this study, the effect of board type (unmodified vs. modified) on the surface roughness of the composites was examined. The results of the surface roughness values (R_a_, R_z_ and R_max_) are presented in Table 3. Statistical analysis was also shown in Table 4. According to these results, composite having MAPE coupling agent provided smoother surfaces than ones without MAPE coupling agent (P<0.0004). It thus appears that treatment with MAPE coupling agent increased the smoothness of the composite surfaces. It is believed that improvement in the adhesion between the wood flour and plastic matrix hindered removal of larger material chunks upon sanding and resulted in better surface properties. Similar findings were also reported by Gupta *et al*. [15]

**Figure 1.**
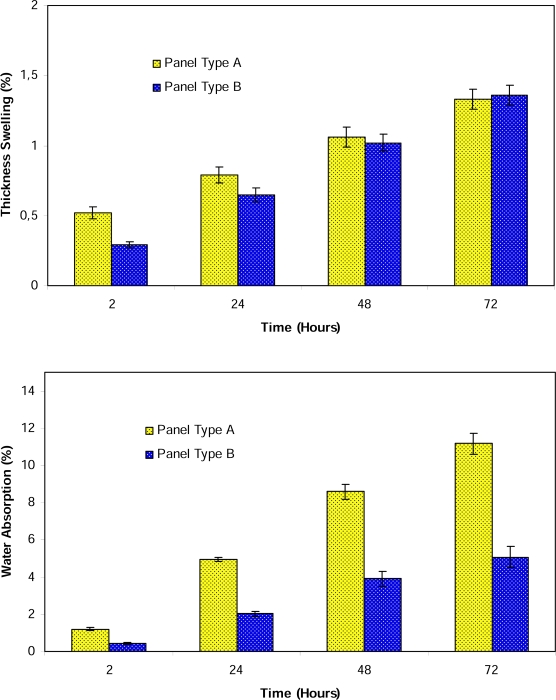
Thickness swelling values and water absorption values of unmodified (A) and MAPE modified (B) HDPE based wood plastic composites.

This study also investigated the effect of board type (unmodified vs. modified) and water soaking time (testing time) on the thickness swelling-water absorption properties of the composites. Thickness swelling and water absorption values of the composites were presented in Figure 1. Statistical analysis was also shown in Table 5. When coupling agent was added into the samples, their thickness swelling values were reduced up to the 24 h soaking time. However, there was no statistical difference between unmodified and modified samples after 24 h soaking as both composites provided similar thickness swelling values.

**Figure 2.**
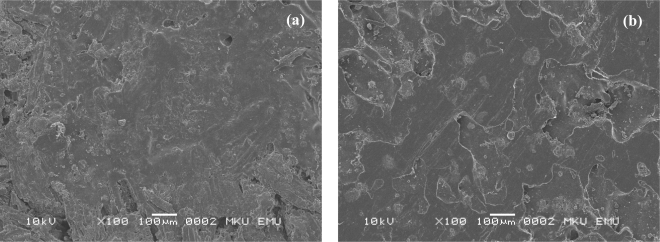
SEM micrographs of (a) 72 hours water soaked unmodified and (b) MAPE modified HDPE based wood plastic composites.

In the case of water absorption, on the other hand, MAPE modification of composites provided better properties compared to unmodified ones. It is believed that in this study the improvement in adhesion between wood flour and plastic matrix through MAPE modification has affected the water absorption properties more than thickness swelling. SEM micrographs of unmodified and MAPE modified samples after 72h water soaking can be seen in Figure 2. Individual wood flours apart from plastic matrix can be seen in Figure 2a. It is believed that water molecules can travel inside the composites between these wood flours resulting in higher weight of samples and higher water absorption values.

### 3.2. Adhesion strength, abrasion and scratch resistance and gloss properties of coated HDPE based wood plastic composites

The effect of board type (unmodified vs. modified) and coating type (polyurethane lacquer vs. cellulose varnish) on the adhesion strength, abrasion and scratch resistance and gloss measurements were also investigated. Summary of the results and statistical analyses were presented in Table 6 and Table 7, respectively.

**Figure 3.**
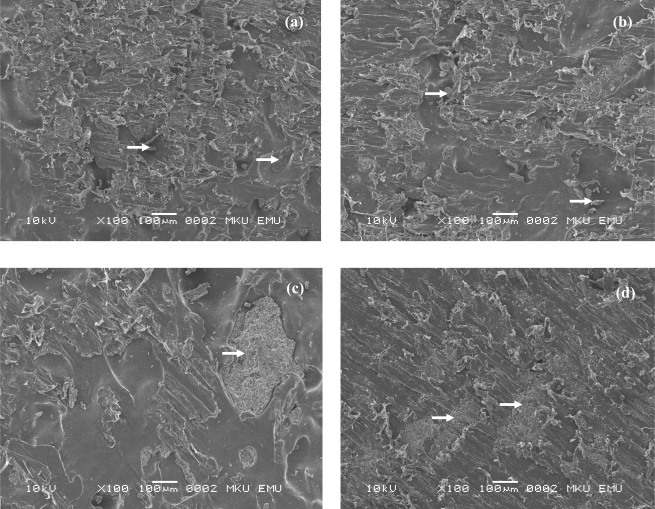
SEM micrographs of the a) unmodified HDPE based wood plastic composites with cellulosic varnish, b) MAPE modified composites with cellulosic varnish, c) unmodified composites with polyurethane varnish, d) MAPE modified composites with polyurethane varnish.

Adhesion strengths of the coatings were determined using the pull-off tests. Both unmodified and MAPE modified composites showed similar and quite poor adhesion strengths. Even though MAPE modified samples and cellulosic varnish coated ones provided slightly higher adhesion strength over unmodified samples and polyurethane lacquer coated samples, respectively, ANOVA tests showed no statistically significant difference among the groups. It appears that plastic matrix prevented both coating systems from penetrating into the samples, so the coating remained on the surface. Coating remaining on the samples can be seen on SEM micrographs (Figure 3). All micrographs were taken at the same magnification and remaining on the surface of the samples after adhesion test were bigger for polyurethane lacquer coated samples compared to cellulosic varnish coated ones. There were openings between coating pieces and samples indicating poor adhesion. However, on the micrographs of the MAPE modified samples, there was small remaining of the coatings on the sample surfaces pointing out little improvement of adhesion.

The results of abrasion resistance and statistical analyses of the results were also presented in Tables 6 and 7, respectively. Statistical analysis showed that there was a significant effect of board type (unmodified vs. MAPE modified) on the abrasion resistance of the composites (P<0.0002). Composites produced with MAPE coupling agent had higher abrasion resistance values compared to unmodified ones regardless of coating type. This could be due to smooth surface obtained after MAPE modification of the samples. Similar findings were also observed in other studies [16, 17]. Coating type, on the other hand, had no significant effect on abrasion resistance (P = 0.3671).

The results of scratch resistance and statistical analyses of the results are shown in Tables 6 and 7, respectively. Statistical analysis showed that there was no significant effect of board type (unmodified vs. MAPE modified) on the scratch resistance of the composites (P=0.1531). Coating type, on the other hand, had significant effect on scratch resistance (P<0.0001). Polyurethane lacquer had higher value than the cellulose varnish. This could be resulted from properties of the coating. Similar findings were also observed on other studies [18]. Gloss values of unmodified and MAPE modified composites coated with cellulose varnish and polyurethane lacquer were shown in Table 6. Statistical analysis was also seen in Table 7. Even though MAPE modified composites had slightly higher gloss values, there was no statistically significant difference between composites (P=0.4353). Regardless of board type, on the other hand, gloss values of the polyurethane lacquer coating were significantly higher than those of cellulose varnish coated (P<0.0001).

## 4. Conclusions

The effect of surface quality and thickness swelling-water absorption properties of recycled high density polyethylene (HDPE) composites (MAPE modified and unmodified) was evaluated. MAPE modification of the composites increased the surface smoothness and reduced the water absorption and thickness swelling of the panels. In addition, cellulosic coating and polyurethane lacquer coating were applied to composite surfaces and their adhesion strength, abrasion and scratch resistance, and gloss values were determined. Improvement of adhesion strength between the coating and composite panels was seen on SEM micrographs. Furthermore, abrasion resistance of the composites was improved through MAPE modification. Regardless of board type, higher scratch resistance and gloss values were observed for polyurethane lacquer coated samples compared to those of cellulosic varnish coated ones.

## Figures and Tables

**Table 1.** Description of the manufactured samples.

ID	Recycled HDPE (%)	Eucalyptus wood residue (%)	MAPE (%)
A	50	50	-
B	46	50	4

**Table 2.** Mixture portion of coatings.

Varnish variety	Varnish (portion)	Hardener (portion)	Thinner (portion)
Cellulosic primer coat	100	-	80
Cellulosic top coat	100	-	80
Polyurethane primer coat	100	50	20
Polyurethane top coat	100	25	80

**Table 3.** Surface roughness of the produced HDPE based wood plastic composites.

*ID*	*Surface roughness (μ)*		
	*R_a_*	*R_z_*	*R_max_*
A	14.91 A^1^	92.31 A	114.93 A
	(4.44) 2	(16.47)	(17.15)
B	8.28 B	63.33 B	89.85 B
	(2.06)	(13.74)	(25.91)

1 The different capital letters show statistically different groups.

2 The value in parenthesis is the standard deviation.

**Table 4.** Analysis of variance (ANOVA) for surface properties (R_a_, R_z_, R_max_) of HDPE based wood plastic composites.

*Dependent Variable*	*Source of variation*	*SS*	*DF*	*MS*	*F*	*P*
R_a_	Board Type	220.12	1	220.12	18.39	0.0004
	Pure Error	215.46	18	11.97		
	Total	435.58	19			
R_z_	Board Type	4200.65	1	4200.65	18.26	0.0005
	Pure Error	4140.19	18	230.01		
	Total	8340.84	19			
R_max_	Board Type	3145.78	1	3145.78	6.52	0.0002
	Pure Error	8685.34	18	482.52		
	Total	11831.13	19			

**Table 5.** Analysis of variance (ANOVA) for thickness swelling - water absorption properties of HDPE based wood plastic composites.

*Dependent Variable*	*Source*	*SS*	*DF*	*MS*	*F*	*P*
Thickness	Model	5.09	7	0.73	55.72	<0.0001
Swelling	Board Type	0.090	1	0.090	6.93	0.0130
	Testing Time	4.91	3	1.64	125.26	<0.0001
	Interaction	0.095	3	0.032	2.43	0.0833
	Pure Error	0.42	32	0.013		
	Total	5.51	39			
Water	Model	41.12	7	5.87	220.37	<0,0001
Absorption	Board Type	7.82	1	7.82	293.36	<0.0001
	Testing Time	33.21	3	11.07	415.30	<0.0001
	Interaction	0.089	3	0.030	1.11	0.3598
	Pure Error	0.85	32	0.027		
	Total	41.97	39			

**Table 6.** Adhesion strength, abrasion and scratch resistance, and gloss properties of the produced HDPE based wood plastic composites.

*Board Type*	*Varnish Type*	*Adhesion Strength (N/mm^2^)*	*Abrasion Resistance (rpm)*	*Scratch Resistance (N)*	*Gloss (°)*
A	Cellulosic	0.157 A^1^ (0.04) 2	20.50 A (4.65)	0.62 A (0.4)	30.08 A (1.35)
A	Polyurethane lacquer	0.155 A (0.03)	19.90 A (5.53)	0.90 B (0.03)	41.39 B (5.74)
B	Cellulosic	0.161 A (0.04)	36.30 B (9.80)	0.64 A (0.05)	30.91 A (0.89)
B	Polyurethane lacquer	0.158 A (0.04)	31.70 B (3.65)	0.94 B (0.05)	43.75 B (11.31)

1 The different capital letters show statistically different groups.

2 The value in parenthesis is the standard deviation.

**Table 7.** Analysis of variance (ANOVA) for adhesion strength, abrasion and scratch resistance, and gloss properties of produced HDPE based wood plastic composites.

*Dependent Variable*	*Source*	*SS*	*DF*	*MS*	*F*	*P*
Adhesion	Model	3.275E-004	3	1.092E-004	0.066	0.9775
Strength	Board Type	2.500E-006	1	2.500E-006	1.512E-003	0.9692
	Varnish Type	1.225E-004	1	1.225E-004	0.074	0.7870
	Interaction	2.025E-004	1	2.025E-004	0.12	0.7284
	Pure Error	0.060	36	1.653E-003		
	Total	0.060	39			
Abrasion	Model	1010.20	3	336.73	8.58	0.0013
Resistance	Board Type	952.20	1	952.20	24.28	0.0002
	Varnish Type	33.80	1	33.80	0.82	0.3671
	Interaction	24.20	1	24.20	0.68	0.4437
	Pure Error	627.60	16	39.22		
	Total	1637.80	19			
Scratch	Model	0.43	3	0.14	70.92	<0,0001
Resistance	Board Type	4.500E-003	1	4.500E-003	2.25	0.1531
	Varnish Type	0.42	1	0.42	210.25	<0.0001
	Interaction	5.000E-004	1	5.000E-004	0.25	0.6239
	Pure Error	0.032	16	2.000E-003		
	Total	0.46	19			
Gloss	Model	1489.35	3	496.45	12.15	<0.0001
	Board Type	25.44	1	25.44	0.62	0.4353
	Varnish Type	1458.06	1	1458.06	35.68	<0.0001
	Interaction	5.85	1	5.85	0.14	0.7074
	Pure Error	1471.32	36	40.87		
	Total	2960.67	39			
